# MicroRNA-29a modulates axon branching by targeting doublecortin in primary neurons

**DOI:** 10.1007/s13238-014-0022-7

**Published:** 2014-02-18

**Authors:** Hanqin Li, Susu Mao, Haitao Wang, Ke Zen, Chenyu Zhang, Liang Li

**Affiliations:** 1 Jiangsu Engineering Research Center for MicroRNA Biology and Biotechnology, State Key Laboratory of Pharmaceutical Biotechnology, Nanjing University School of Life Sciences, Nanjing, 210093 China; 2 Department of Pharmacy, 306th Hospital of PLA, Beijing, 100101 China

**Keywords:** miR-29a, doublecortin, glutamate receptor, mature neurons, axon branching

## Abstract

MicroRNAs (miRNAs) are endogenously expressed small, non-coding transcripts that regulate protein expression. Substantial evidences suggest that miRNAs are enriched in central nervous system, where they are hypothesized to play pivotal roles during neural development. In the present study, we analyzed miRNAs expression in mice cerebral cortex and hippocampus at different developmental stages and found miR-29a increased dramatically at postnatal stages. In addition, we provided strong evidences that miR-29a is enriched in mature neurons both *in vitro* and *in vivo*. Further investigation demonstrated that the activation of glutamate receptors induced endogenous miR-29a level in primary neurons. Moreover, we showed that miR-29a directly regulated its target protein Doublecortin (DCX) expression, which further modulated axon branching in primary culture. Together, our results suggested that miR-29a play an important role in neuronal development of mice cerebrum.

**Electronic supplementary material:**

The online version of this article (doi:10.1007/s13238-014-0022-7) contains supplementary material, which is available to authorized users.

## Introduction

MiRNAs are approximately 21 nucleotide non-coding transcripts that are derived from hairpin precursors, which regulate protein expression by base pairing to the 3′ untranslated region (UTR) of mRNAs (Chen et al., 2008). Several works show that miRNAs are enriched in central nervous system and emerging evidence have suggested the pivotal roles of miRNAs in neural development (Ji et al., 2013; Shu et al., 2013). Mutations in Drosophila Ago1 cause a severe loss of all types of neurons and glial cells while dicer deficient mice display abnormal neurogenesis and gliogenesis in the developing central nervous system (Kataoka et al., 2001; Kawase-Koga et al., 2009). There are also studies indicating the significant role of specific miRNA in neural development. For example, miR-132 modulates the dopamine neuron differentiation while miR-124 regulates neurite outgrowth during neuronal differentiation (Yang et al., 2012; Yu et al., 2008). Together, these data support the notion that miRNAs modulate cell differentiation, maturation as well as survival in central nervous system.

To investigate the function of miRNAs in the development of mice cerebrum, we screened miRNAs expression in mice cerebrum at different developmental stages. Interestingly, we found miR-29a changed most dramatically among all those miRNAs that increased during cerebrum development. MiR-29 family is consisted of three mature members, miR-29a, miR-29b, and miR-29c, which are encoded by two genome clusters (Wang et al., 2013). Recent studies have demonstrated that miR-29 family is associated with the pathogenesis of sporadic Alzheimer’s disease by targeting BACE and NAV3 (Bettens et al., 2009; Shioya et al., 2010). Further investigation indicates that miR-29b has anti-apoptotic effect in mature neuron by targeting BH3-only (Kole et al., 2011). However, the role of miR-29 family during neuronal development remains obscure, which still needs further investigation.

In the present study, we demonstrated that miR-29a, which highly expresses in mature neurons, is up-regulated both in cortex and hippocampus during cerebrum development especially in postnatal stages. Besides, we provided evidences that activation of glutamate receptors contribute to the increase of miR-29a in primary neurons, suggesting the possible association of neural activity and the increase of miR-29a. Moreover, our data revealed that DCX, a protein that is reported to affect axon branching (Bilimoria et al., 2010), is a direct target of miR-29a. Further functional analysis showed that miR-29a increased axon branching via repressing DCX expression. Taken together, these data indicate that miR-29a plays an important role in neuronal development of mice cerebrum.

## Results

### Screening miRNAs expression levels in mice cerebral cortex at different developmental stages

To investigate the role of miRNAs in the development of mice cerebrum, we first analyzed the miRNAs levels in the mice cortex at different stages (E12.5, E15.5, E18.5 and P60) by miRNAs array. Here we mainly focused on the increased miRNAs which are selected based on the following two criteria: (1) the minimum copy number of miRNA at P60 is above 2000; (2) the fold change of miRNA between P60 and E12.5 is more than 5. Among all those selected miRNAs, miR-29a increased most significantly in P60 mice cortex (Table 1). Meanwhile, the copy number of miR-29b and miR-29c is less than 300 at the stage of P60 (data not shown). Surprisingly, miR-29a did not change significantly during E12.5–E18.5 stages while it increased drastically (>70 fold) during E18.5–P60 stages. To further understand the correlation between miR-29a and cerebrum development, we analyzed miR-29a expression both in cortex and hippocampus by Q-RTPCR. We found that cortical miR-29a showed a time-dependent increase exclusively in postnatal stages while miR-138 increased gradually during the entire developmental stages analyzed (E12.5–P60), which is consistent with the miRNAs array analysis (Fig. 1A). Similarly, the level of miR-29a in hippocampus began to increase at P7 while the level of miR-138 gradually increased during the whole stages (E18.5–P60) (Fig. 1B). We also compared the expression levels of miR-29 family in cortex and hippocampus of P60 mice by Q-RTPCR and demonstrated that miR-29a concentration is relatively higher than that of the other two family members (Fig. S1).

**Table 1:** Copy number of miRNAs that increased significantly during the cerebrum development

	Copy number at different developmental stages				Ratio
	E12.5	E15.5	E18.5	P60	P60/E12.5
miR-22	650.86	753.18	1135	7193.4	11.05
miR-23a	1249.1	1817.3	1482.8	6461.2	5.17
miR-24	3396.8	5393	5354	20931	6.16
miR-29a	61.68	61.58	73.86	5122.3	83.05
miR-31	228.11	245.63	352.49	3118.1	13.67
miR-128	945.99	1333.8	4568.2	7894.6	8.35
miR-132	510.18	901.66	1727.1	16807	32.94
miR-138	1713.5	4395.6	6829.2	23379	13.64
miR-139-5p	359.66	484.26	636.85	4870.7	13.54
miR-143	285	291.6	411.99	2356.9	8.27
miR-145	640.83	638.49	821.15	4777.1	7.45
miR-221	747.36	478.43	901.21	5651.1	7.56
miR-222	463.46	426.43	783.48	9207.9	19.87

**Figure 1:**
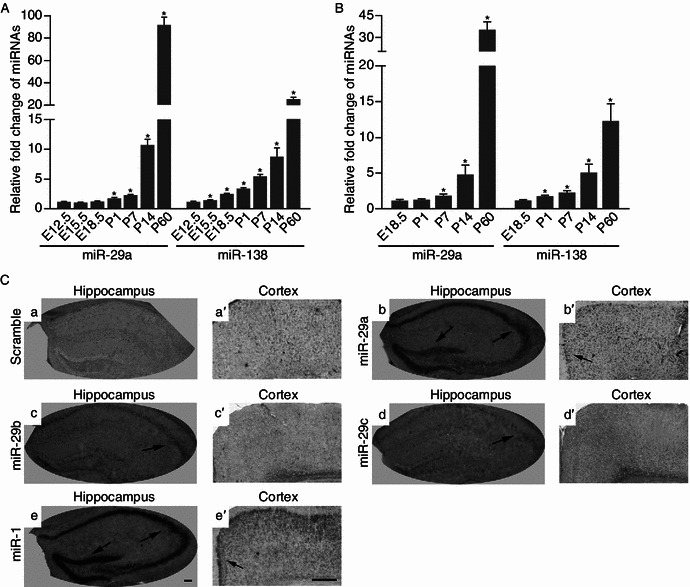
**Expression of miR-29a in mice cortex and hippocampus**. (A) Relative expression level of miR-29a and miR-138 (U6 is used as internal control) in cortex of different developmental stages, *, *P* < 0.01 compared to the E12.5 stage in each group (*n* = 5); (B) relative expression level of miR-29a and miR-138 in hippocampus of different developmental stages, *, *P* < 0.01 compared to the E18.5 stage in each group (*n* = 5); (C) *in situ* hybridization of different members of miR-29 family in hippocampus (a–e) and cortex (a′–e′) (*n* = 3), miR-124 is used as positive control, arrows indicate neurons with positive signal in cortex or hippocampus, scale bar = 200 μm

### Expression pattern of miR-29 family in mice cerebrum

To elucidate the expression pattern of miR-29 family in mice cerebrum, we examined three members of miR-29 family including miR-29a, miR-29b and miR-29c by *in situ* hybridization. Our results demonstrated that miR-29a is most intensively expressed in hippocampus neurons while miR-29b and miR-29c showed much weaker signals (Fig. 1C, a–e). Besides, miR-29a is also expressed in cortical neurons, while miR-29b and miR-29c barely showed any positive signals (Fig. 1C, a′–e′). These results are coincident with our Q-RTPCR analysis (Fig. S1). Nevertheless, using lung tissue as positive control, we demonstrated that both miR-29b and miR-29c show relatively strong signals in the mice lung tissue compared with that in cortex and hippocampus (Fig. S2). Importantly, we also analyzed miR-29a expression pattern in dentate gyrus (DG) area at different stages. The signal appeared at P7 and increased at P14 and P60, which is consistent with our Q-RTPCR analysis (Fig. 2A, a–c). Furthermore, the expression of miR-29a in DG area began from the outside of granule layer (P7) while gradually spread to the inside (P60), which is similar to the neuron maturation pattern marked by NeuN (mature neuronal marker) (Fig. 2A, d–f) (Snyder et al., 2012). Together, these data provide evidence indicating that miR-29a is highly expressed and functions in the development of cerebrum, especially in mature neurons.

**Figure 2:**
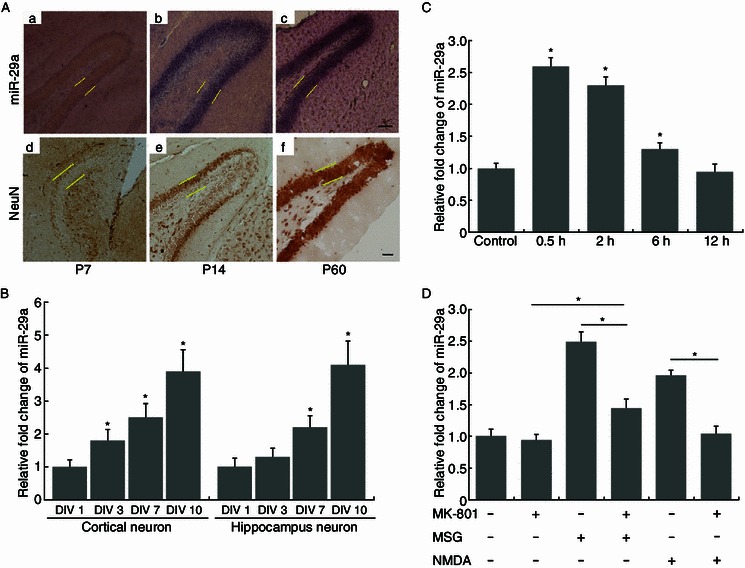
**Expression of miR-29a in neurons under different circumstances**. (A) Expression pattern of miR-29a (purple) in DG area of hippocampus at different postnatal stages (a–c), expression pattern of NeuN (brown) in DG area of hippocampus at different postnatal stages (d–f), granule cell layers are indicated between two yellow lines (*n* = 3), scale bar = 50 μm; (B) relative expression level of miR-29a in primary neuron at different cultured stages, *, *P* < 0.01 compared to DIV 1 in each group (*n* = 5); (C) relative expression level of miR-29a in primary cortical neurons at different time points after the stimulation of 10 μmol/L GSM for 15 min, *, *P* < 0.01 compared to control (*n* = 3); (D) relative expression level of miR-29a in primary cortical neurons at 2 h after indicated treatments, *, *P* < 0.01 (*n* = 3)

### Activation of glutamate receptors increases the level of miR-29a in primary neurons

We have observed the up-regulation of miR-29a during cerebrum development exclusively at postnatal stages (Fig. 1A and 1B). In addition, we also examined the miR-29a level in cultured neuron from cortex or hippocampus at different time points and showed that miR-29a increased as neurons became mature (Fig. 2B). Therefore, it is interesting for us to elucidate the mechanism underlying the regulation of miR-29a expression. We investigated the association between neural activity and miR-29a expression by assessing the level of miR-29a in cortical neurons after glutamate treatment (10 μmol/L, 15 min) and found that miR-29a is significantly increased since 0.5 h after the stimulation. Such increase lasted for 6 h and miR-29a returned to the basal level after 12 h (Fig. 2C). Furthermore, we found that pretreatment of MK-801 (NMDA receptor antagonist) significantly blocked the increase of miR-29a induced by glutamate (Fig. 2D). Similarly, DNQX (AMPA/kainate receptor antagonist) also inhibited such increase and exhibited linear effect when used with MK-801 together (Fig. 2D). These data indicate that the activation of glutamate receptors may contribute to the increase of miR-29a in neurons during cerebrum maturation.

### DCX is a direct target of miR-29a

Through bioinformatics screening, we found that the sequence of miR-29a matched well with 3′ UTR of DCX mRNA (Fig. 3A). DCX is a microtubule-associated protein expressed by neuronal precursor cells and immature neurons, which is reported to modulate axon branching (Bilimoria et al., 2010; Spampanato et al., 2012). In addition, the targeted sequence in DCX mRNA that matched with the seed sequence of miR-29a is highly conservative in many species, indicating the regulation of DCX expression by miR-29a is quite universal. We further evaluate the protein level of DCX in both cortex and hippocampus at different developmental stages. In embryonic stages, DCX level slightly increased in cortex (E18.5) while did not change a lot in hippocampus. However, in postnatal stages, DCX expression significantly decreased both in cortex and hippocampus at P7 (Fig. 3B–D), which is negatively correlated with the change of miR-29a level in cortex and hippocampus (Fig. 1A and 1B). We also demonstrated that the expression patterns of DCX and miR-29a in DG area are mutually complementary (Figs. 3E and 2A). Moreover, we analyzed the expression of DCX during the neuron maturation *in vitro* and found the level of DCX showed a time-course decrease, which is negatively correlated to the level of miR-29a (Fig. 3F and 3G). To prove that DCX is a direct target of miR-29a we conducted luciferase assay and found that miR-29a markedly reduce the translational efficiency of reporter gene tagged with the 3′UTR of DCX mRNA containing the putative miR-29a-binding sites while mutations in these sites significantly blocked the inhibitory effect (Fig. 3H). Next, through gain- and loss-of-function approaches, we evaluated the effect of miR-29a on DCX expression in primary neurons. Over-expression of miR-29a resulted in a remarkable decrease of DCX protein level in primary neurons (Fig. 3I–K). Meanwhile, antago-miR-29a reduced cellular miR-29a level and increased DCX protein expression in cultured neurons (Fig. 3L–N). Together, these evidences support the notion that miR-29a inhibits DCX expression by directly targeting its 3′UTR of mRNA.

**Figure 3:**
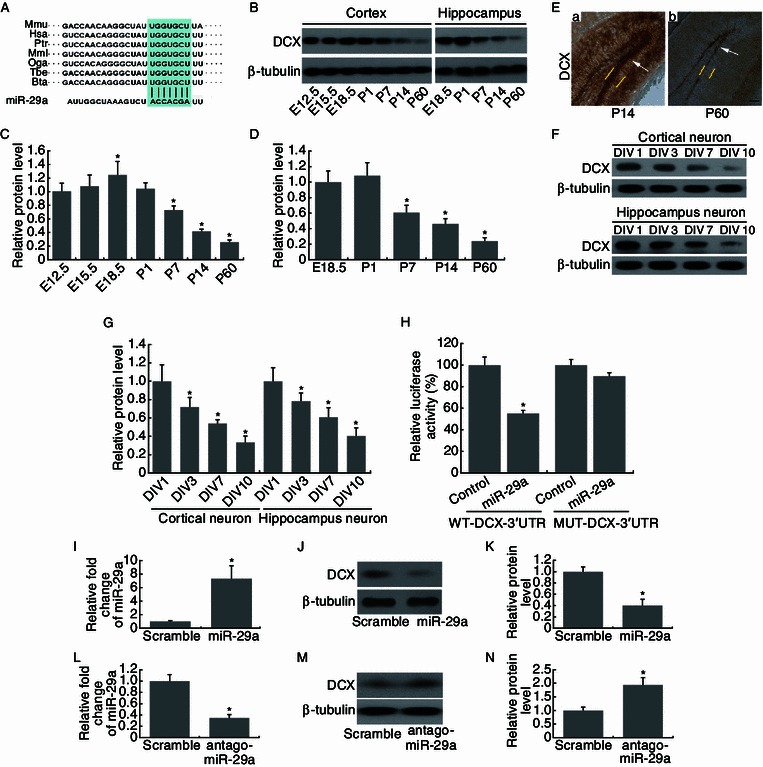
**DCX is a direct target of miR-29a**. (A) Seed sequence and alignment of the miR-29a binding sites in the 3′UTRs of DCX mRNAs from different species, the predicted base-pairing of miR-29a with target recognition seed sequence is shown in colored; (B) Western-blot of DCX (β-tubulin is used as internal control) in cortex (left panel) or hippocampus (right panel) at different developmental stages indicated (*n* = 3); (C and D) quantification of DCX protein in cortex (C) or hippocampus (D) at different developmental stages indicated (*n* = 3); (E) expression pattern of DCX (brown, arrows) in DG area of hippocampus at different postnatal stages, granule cell layers are between two yellow lines (*n* = 3), scale bar = 50 μm; (F and G) Western-blot and quantification of DCX protein in primary neurons derived from cortex and hippocampus at different time points (*n* = 3); (H) relative luciferase activity measured in different groups of 293T cells, *, *P* < 0.01 compared to control (*n* = 3); (I) relative level of miR-29a in primary cortical neurons transfected with miR-29a mimics, *, *P* < 0.01 compared to scramble (*n* = 3); (J and K) Western-blot and quantification of DCX protein in primary cortical neurons treated as in G, *, *P* < 0.01 compared to scramble (*n* = 3); (L) relative level of miR-29a in primary cortical neurons transfected with antago-miR-29a, *, *P* < 0.01 compared to scramble (*n* = 3); (M, N) Western-blot and quantification of DCX protein in primary cortical neurons treated as in J, *, *P* < 0.01 compared to scramble (*n* = 3)

### MiR-29a increases axon branching in primary cortical neuron via targeting DCX

Since DCX is reported to restrict axon branching we further investigated the role of miR-29a in axon growth of primary neurons. We over-expressed miR-29a in cortical neurons at DIV 3 and found it triggered exuberant branching of axons at DIV 7 (Fig. 4A and 4B). On the contrary, inhibition of endogenous miR-29a by antago-miR resulted in long axons bearing few or no branches of primary neurons (Fig. 4C and 4D). In addition, to elucidate the role of DCX in miR-29a induced axon branching, we specifically knocked down the endogenous DCX protein by RNAi (Fig. 4E and 4F). In the morphology assay, we found that DCX knockdown also induced excessive axonal branches compared with control neurons (Fig. 4G and 4H). Furthermore, we also over-expressed DCX (without miR-29 binding site) and demonstrated an effective inhibition of the axon branching induced by miR-29a over-expression (Fig. 5). These results indicate that miR-29a may increase axon branching in primary neuron by repressing DCX expression.

**Figure 4:**
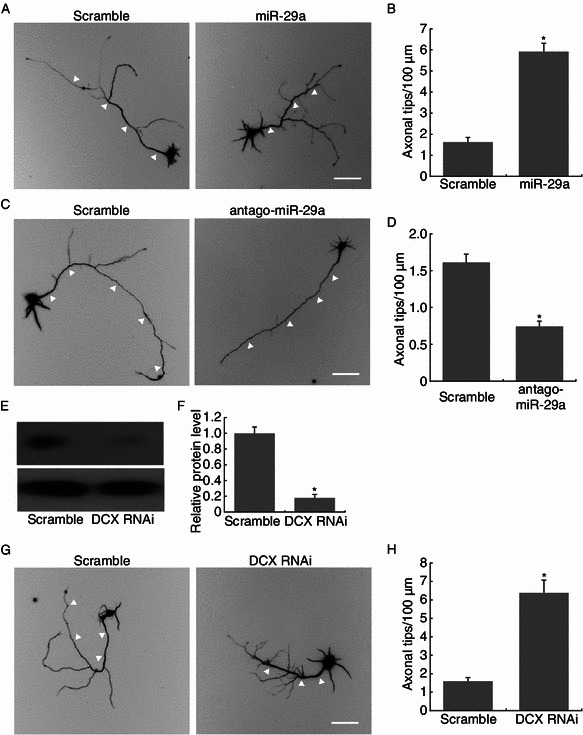
**Effects of miR-29a on axon branching of primary cortical neurons**. (A) Cortical neurons transfected with scramble or miR-29a mimics together with the eGFP expression plasmid were fixed 4 d after transfection and subjected to immunocytochemistry using the eGFP antibody. In all images of this type, arrowheads indicate primary axons, scale bars = 50 μm; (B) quantification of axon branching of cortical neurons treated in A, *, *P* < 0.01 compared to scramble (*n* = 20); (C) cortical neurons transfected with scramble or antago-miR-29a together with the eGFP expression plasmid were analyzed as in A, scale bars = 50 μm; (B) quantification of axon branching of cortical neurons treated in C, *, *P* < 0.01 compared to scramble (*n* = 20); (E, F) Western-blot and quantification of DCX protein in cortical neurons treated with scrambles or DCX RNAi (*n* = 3); (G) cortical neurons transfected with scrambles or DCX RNAi together with the eGFP expression plasmid were analyzed as in A, scale bars = 50 μm; (H) quantification of axon branching of cortical neurons treated in G, *, *P* < 0.01 compared to scramble (*n* = 20)

**Figure 5:**
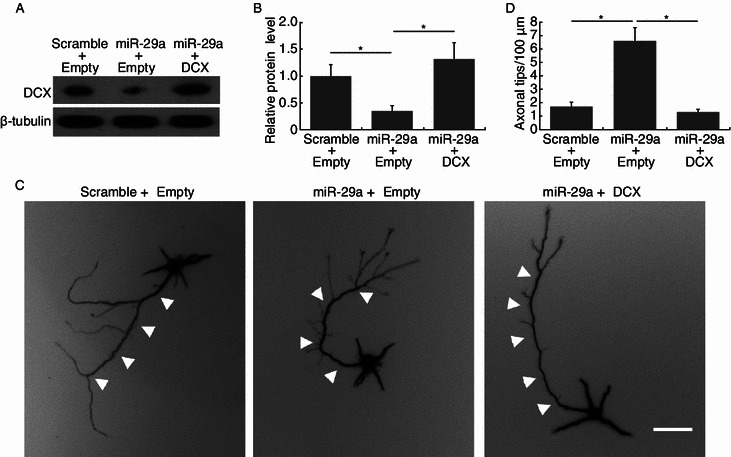
**Over-expression of DCX rescues the axon branching phenotype induced by miR-29a**. (A and B) Western-blot and quantification of DCX protein in cortical neurons under different treatment indicated (*n* = 3); (C) different groups of cortical neurons transfected together with the eGFP expression plasmid were analyzed as in Fig. 4A, scale bars = 50 μm; (D) quantification of axon branching of cortical neurons treated in C, *, *P* < 0.01 compared to scramble (*n* = 20)

## Discussion

MiRNAs are small, non-coding transcripts that functioned in many tissues by repressing target gene expression (Chen et al., 2012). Circumstantial evidences suggest that miRNAs play a crucial role in the central nervous system (Ji et al., 2013). To elucidate the role of miRNAs in cerebrum development, we analyzed the miRNAs expression pattern in mice cerebrum and found several miRNAs that increase significantly during different developmental stages, among which miR-29a changed most significantly. The expression level of miR-29a showed no significant differences until postnatal stages when it increased dramatically both in cortex and hippocampus, indicating miR-29a may be associated with the process of neural maturation.

Since it is reported that miR-29 expressed more strongly in cultured astrocyte than in neuron (Smirnova et al., 2005), we investigated the expression pattern of miR-29 *in vivo* by *in situ* hybridization. Our results demonstrated that miR-29a mainly expressed in mature neuron in cerebrum while miR-29b and miR-29c showed relatively weak signals. These discrepancies may be the result of differences between *in vitro* and *in vivo* as well as the techniques with different sensitivity used for detecting miRNAs. In addition, we provided evidence that the expression pattern of miR-29a in DG area is similar to that of NeuN (mature neuronal marker), which suggested that miR-29a is increased during neuron maturation. Furthermore, we showed a time-course increase of miR-29a expression in primary neurons. Together, these data indicate that miR-29a may play an important role in mature neurons.

Since neural stimulation helped to fine-tune the process of neuronal maturation (He et al., 2010), we further investigated the association between miR-29a expression and neural activity. Our results demonstrated that activation of glutamate receptors induced the endogenous level of miR-29a in primary neurons indicating that neural activity may modulate the expression of miR-29a.

Through bioinformatics screening and luciferase assay, we searched and verified one target gene of miR-29a—DCX. DCX initially expressed in neuronal precursor cells and immature neurons (E12.5) while it began to down-regulate around P7, which is coincident with the time that miR-29a aroused in cerebrum (Spampanato et al., 2012). Immunostaining results also revealed that DCX expressed in a complementary pattern compared with the expression of miR-29a in DG area. Besides, protein analysis also demonstrated that miR-29a inhibited DCX translation in primary neurons.

As a target protein of miR-29a, DCX is a microtubule associated protein that stabilized microtubules (Cohen et al., 2008). There is evidence that GSK3β/DCX signaling pathway restricts axon branching (Bilimoria et al., 2010). In addition, neural activity that has been proved to affect miR-29a expression is also reported to regulate axon development (Uesaka et al., 2005). Therefore, it is necessary for us to evaluate the effect of miR-29a on axon growth. Our data suggested that miR-29a triggered excessive axon branching while blocking endogenous miR-29a resulted in the opposite effect. Moreover, DCX RNAi also promoted axon branching of cortical neurons, which is similar to the effect of miR-29a over-expression. MiR-29 has multiple targets that may affect neuronal growth (Govek et al., 2005; Wang et al., 2013). To further confirm the role of DCX in the miR-29a regulated axon branching, we over-expressed DCX and found it effectively rescued the axon branching phenotype induced by miR-29a over-expression. Together, these results suggest that miR-29a modulated axon branching via targeting DCX expression.

Taken together, our data demonstrate that activation of glutamate receptors increased the expression of miR-29a, which further promoted axon branching in the maturation of cerebral neurons by targeting DCX. Whether miR-29a-DCX regulation pathway is involved in the neural activity dependent axon growth *in vivo* needs further investigation.

## Material and Methods

### Primary culture

Animal care and procedures were performed in accordance with the Laboratory Animal Care Guidelines approved by Model Animal Research Center of Nanjing University. Primary neurons were cultured from cortex of E16.5 mice as described previously (David et al., 2007). Neurons were seeded at 3 × 10^5^ per well in 12 well plate for biochemistry analysis, or at 1 × 10^4^ per well for immunofluorescent staining and morphological observation. Half medium was refreshed every 2 d.

Cultured neurons (DIV 3) were transfected with miR-29a mimics (Genepharma), antago-miR-29a (RiboBio Inc) or DCX RNAi (Genepharma, described in (Ramos et al., 2006)) together with eGFP reporter plamid using lipofectamine 2000 (Invitrogen) according to the manufacturer’s protocol. Cells were harvested 24 h later for RNA analysis and 48 h later for protein analysis. For morphological study, cells were fixed 4 d after transfection.

Neurons (DIV 3) were subjected to the stimulation of MSG (10 μmol/L) for 15 min. After that RNA samples were collected at the time indicated. For the inhibition of glutamate receptor, cells were pretreated with MK-801 (10 μmol/L, Sigma) or/and DNQX (10 μmol/L, Sigma) for 2 h before subjecting to MSG stimulation.

### RNA and protein quantification

Mice cortex and hippocampus were dissected from C57BL/6 J mouse at different stages indicated. Total RNA was purified from the cultures or tissues with TRIZOL (Invitrogen) according to the manufacturer’s instructions. The RNA purity was determined by the OD_260_/OD_280_ ratio, and the concentration was calculated based on OD_260_. 50 μg of total RNA of cortexes from different stages (E12.5, E15.5, E18.5 and P60) were sent to the Capitalbio Corporation for miRNAs array analysis.

Q-RTPCR was carried out using TaqMan microRNA probes (Applied Biosystems). Briefly, 2 μg of total RNA was reverse-transcribed to cDNA using AMV reverse transcriptase (TaKaRa) and stem-loop RT primers (Applied Biosystems). Real-time PCR was performed using a TaqMan PCR kit on an Applied Biosystems 7300 Sequence Detection System (Applied Biosystems). All reactions, including the no-template controls, were run in triplicate. After the reactions, the *C*T values were determined using fixed-threshold settings. Upon the evaluation of each miRNA’s level, the expression level of U6 was used as internal control.

Cultures or tissues were both lysed in 50 mmol/L Tris, pH 7.5, 150 mmol/L NaCl, 2 mmol/L EDTA, and 1% Triton X-100. The protease inhibitors aprotinin, pepstatin, leupeptin, as well as the reducing agent dithiothreitol were added to this buffer before cell lysis. Lysates were cleared of insoluble material by spinning at maximum speed on a tabletop centrifuge, boiled in sample buffer, and examined using standard SDS-PAGE, followed by Western blotting. The antibody used were DCX (Abcam) and β-tubulin (Abcam).

### *In situ* hybridization

This method was adjusted based on the protocol described by Obernosterer et al (Obernosterer et al., 2007). Brains were fixed in 4% paraformaldehyde overnight at 4°C and cryo protected in 15% sucrose followed by 30% sucrose overnight each. Cryostat section (20 μm) were cut and mounted onto Superfrost Gold Plus slide (Fisher). Then sections were post-fixed in 4% paraformaldehyde followed by washed in PBS for three times and 75% alcohol for one time, dried in RT, and incubated in hybridization buffer (DEPC H_2_O with 50% de-ionized formamide (*v/v*), 5× SSC, 0.2 mg/mL yeast tRNA, 0.5 mg/mL herring sperm DNA, 10% dextran sulfate (*w/v*), 1/1000 Tween-20) with a concentration of 10 nmol/L for digoxigenin-labeled LNA probes (Exiqon) overnight at 50°C in a humidified chamber. Following hybridization, slides were washed in 0.1× SSC at 55°C for 3 times, 30 min each, then blocked in 10% FBS in TBS (pH = 7.5) for 1 h. AP-conjuncted-anti-digoxigenin antibody (Roche) was incubated with slides overnight at final concentration of 1:1000. NBT/BCIP kit (Invitrogen) was used to do the color reaction.

### Plasmid construction and luciferase assay

Part of mmu-DCX 3′UTR containing the predicted target sequence was obtained from cortex of P1 mouse using RT-PCR with primers as indicated. Forward primer 5′-AAGATGATAGGCTAAATCAAAGCC-3′, reverse primer 5′-CAGGATGAATGCTAGACAATACAC-3′. This PCR product was inserted into pMIR-REPORTTM-Luciferase vector (Ambion) after digested with *Hin*dIII and *Sac*I (Takara) to construct WT vector. Mutant vector was generated in Invitrogen by replacing the predicted target region with its reverse sequence (from AAGCACCAATAGCCTTGTTGGTC to CTGGTTGTTCCGATAACCACGAA). The oligonucleotides used for the RT-PCR cloning of DCX without miR-29 binding site: forward primer 5′-ggttccaccaaaatATGg (ATG initiator), reverse primer 5′-TCAcatggaatcgccaag (TGA terminator codon).

293T cell was cultured in DMEM (Gibco) containing 10% FBS (Gibco) and seeded in 24 well plate. 12 h after plating, 0.2 μg of firefly luciferase reporter plasmid, 0.2 μg of β-galactosidase expression vector (Ambion), and equal amounts (20 pmol) of miR-29a mimics or scrambled negative control RNA were transfected into cells with Lipofectamine 2000 as manufacturer’s instructing. The β-galactosidase vector was used as a transfection control. At 24 h post-transfection, cells were analyzed using a luciferase assay kit (Promega).

### Immunochemistry

Primary cortical neurons were briefly washed twice with cold PBS, and then fixed in 4% paraformaldehyde for 10 min at room temperature (RT). Neurons after fixation were washed with cold PBS three times, permeabilized and blocked with 2% BSA and 0.05% Triton X-100 in PBS for 1 h (RT). Next, neurons were incubated with primary antibodies (anti-eGFP, Abcam) in 2% BSA/PBS in a humidified chamber overnight at 4°C and rinsed three times in PBS (3 × 5 min). This was followed by incubation with secondary antibodies (Invitrogen) in 2% BSA/PBS in a light-proof container (2 h at RT).

Slides were washed and blocked with PBS containing 0.3% Triton X-100 and 5% BSA (Sigma), followed with the incubation of primary antibodies (NeuN, Millipore; DCX, Abcam) overnight at 4°C. Sections were washed three times in PBS and then incubated with biotinylated secondary antibody (1:500, Vector Laboratories, Burlingame, California, USA) overnight at 4°C. After that signals were further amplified using peroxidase Vectastain ABC reagent (Vector Laboratories). 3,3′-diaminobenzidine (Sigma) was used for the color reaction.

### Morphological analysis

Images were captured on an Olympus microscope and analyzed using Necromantic software. Axon tips were defined as terminal points of the primary axon and all of its branches.

### Statistical Analysis

Data are presented as sample means and their standard errors. Two-tailed Student’s *t* test was used for comparison of the means between any two groups. Differences were considered significant when *P* < 0.05.

## Electronic supplementary material

Below is the link to the electronic supplementary material.

Supplementary material 1 (PDF 223 kb)
