# Supplementary material for: MicroRNA-29a modulates axon branching by targeting doublecortin in primary neurons
Source: Protein Cell. 2014 Feb 18;5(2):160–9. doi: 10.1007/s13238-014-0022-7 (PMC3956970; doi:10.1007/s13238-014-0022-7)
Supplement: Supplementary file 1 [file 13238_2014_22_MOESM1_ESM.pdf]

Sfigure 1

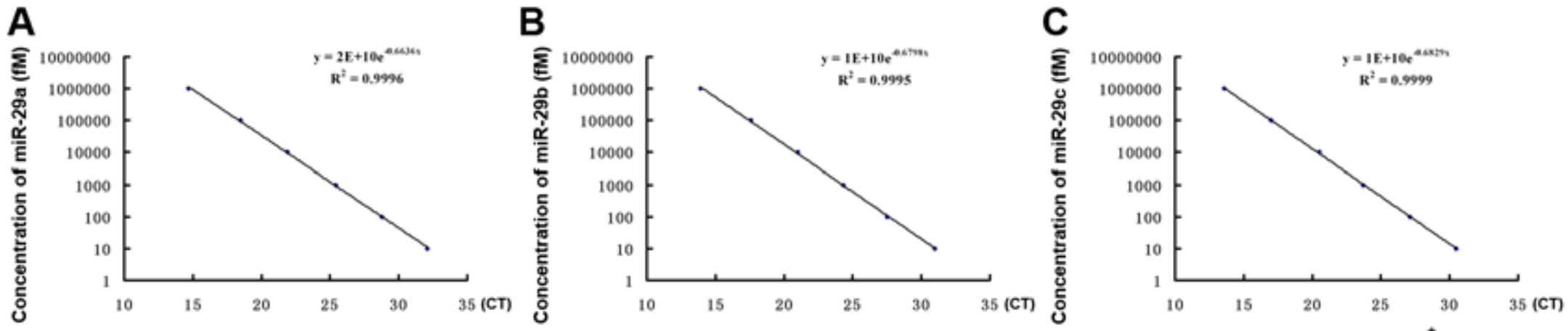

D

	miR-29a		miR-29b		miR-29c	
	CT value	Concentration (pM)	CT value	Concentration (pM)	CT value	Concentration (pM)
Cortex	19.2±0.5	60.7±19.9	19.6±0.4	16.8±4.5	20.1±0.4	11.2±3.0
Hippocampus	17.6±0.3	171.5±34.0	17.9±0.5	53.9±18.1	18.7±0.6	30.1±12.1

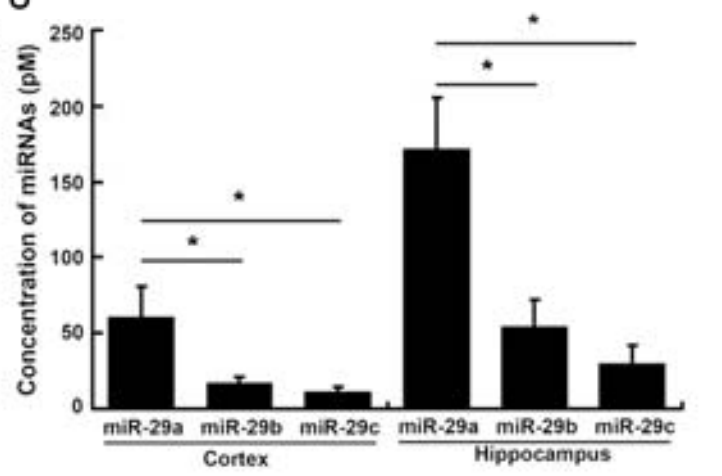

Sfigure 1 (A-C) standard curves derived from different concentration of synthetic mature miR-29a, miR-29b and miR-29c respectively; (D) CT value and calculated concentration of miR-29a/b/c in the RNA samples derived from P60 mice cortex and hippocampus; (E) comparison of the levels of miR-29a/b/c in P60 mice cortex and hippocampus

Sfigure 2

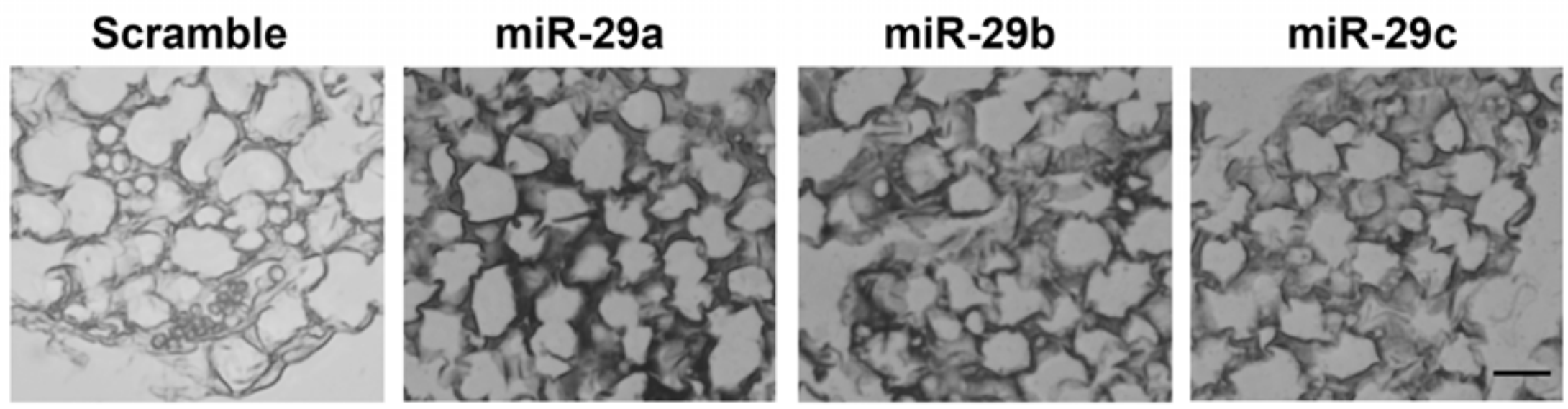

Sfigure 2 *In situ* hybridization of different members of miR-29 family in mice lung tissues as positive controls, Scale bar= 50μm.
